# Web-Based Intervention for Women With Type 1 Diabetes in Pregnancy and Early Motherhood: Critical Analysis of Adherence to Technological Elements and Study Design

**DOI:** 10.2196/jmir.9665

**Published:** 2018-05-02

**Authors:** Marie Berg, Karolina Linden, Annsofie Adolfsson, Carina Sparud Lundin, Agneta Ranerup

**Affiliations:** ^1^ Centre for Person-Centred Care (GPCC), Institute of Health and Care Sciences Sahlgrenska Academy University of Gothenburg Gothenburg Sweden; ^2^ School of Health Sciences Örebro University Örebro Sweden; ^3^ Department of Applied Information Technology University of Gothenburg Gothenburg Sweden

**Keywords:** randomized controlled trial, eHealth, mHealth, case study

## Abstract

**Background:**

Numerous Web-based interventions have been implemented to promote health and health-related behaviors in persons with chronic conditions. Using randomized controlled trials to evaluate such interventions creates a range of challenges, which in turn can influence the study outcome. Applying a critical perspective when evaluating Web-based health interventions is important.

**Objective:**

The objective of this study was to critically analyze and discuss the challenges of conducting a Web-based health intervention as a randomized controlled trial.

**Method:**

The MODIAB-Web study was critically examined using an exploratory case study methodology and the framework for analysis offered through the Persuasive Systems Design model. Focus was on technology, study design, and Web-based support usage, with special focus on the forum for peer support. Descriptive statistics and qualitative content analysis were used.

**Results:**

The persuasive content and technological elements in the design of the randomized controlled trial included all four categories of the Persuasive Systems Design model, but not all design principles were implemented. The study duration was extended to a period of four and a half years. Of 81 active participants in the intervention group, a maximum of 36 women were simultaneously active. User adherence varied greatly with a median of 91 individual log-ins. The forum for peer support was used by 63 participants. Although only about one-third of the participants interacted in the forum, there was a fairly rich exchange of experiences and advice between them. Thus, adherence in terms of social interactions was negatively affected by limited active participation due to prolonged recruitment process and randomization effects. Lessons learned from this critical analysis are that technology and study design matter and might mutually influence each other. In Web-based interventions, the use of design theories enables utilization of the full potential of technology and promotes adherence. The randomization element in a randomized controlled trial design can become a barrier to achieving a critical mass of user interactions in Web-based interventions, especially when social support is included. For extended study periods, the technology used may need to be adapted in line with newly available technical options to avoid the risk of becoming outdated in the user realm, which in turn might jeopardize study validity in terms of randomized controlled trial designs.

**Conclusions:**

On the basis of lessons learned in this randomized controlled trial, we give recommendations to consider when designing and evaluating Web-based health interventions.

## Introduction

### Background

One important component of eHealth research is the evaluation of the use of information and communication technology. This was stated by Eysenbach more than 15 years ago [1] and further critically discussed, focusing on emerging Web-based interventions for patients with chronic illnesses. The issue of using randomized controlled trials (RCTs) as the “gold-standard” research methodology was also highlighted, as was the risk that the control group could become involved with other Web-based applications with similar objectives, thus “contaminating” the design [2]. Since then, the global use of mobile phones, the Internet, and a plethora of related technologies and applications has increased exponentially, and numerous interventions for promoting health and health-related behaviors have been carried out.

Thus, applying a critical perspective to Web-based interventions for persons with long-term and chronic illnesses is of specific importance. However, several methodological challenges exist, for example, issues of recruitment, randomization, fidelity, retention data quality, and degree of adherence [3]. Another matter is how the used technology and its design influence a Web-based intervention. To investigate whether intervention characteristics and persuasive design affect adherence, 83 Web-based health interventions were reviewed by Kelders et al [4]. Through the use of a framework for Persuasive System Design (PSD) [5] and its design principles, they coded persuasive technology elements and analyzed intervention characteristics and adherence. In a multiple regression analysis, they found that users’ adherence to an intervention was predicted by differences in technology and interaction with a counselor. Providing persuasive technology for social support did not affect adherence, although the need to further explore this was stressed [4].

### Objectives

In this paper, we further critically analyze and discuss the challenges of conducting an RCT using Web-based technology, including devices for social support, with special focus on adherence. According to Kelders et al, adherence is defined as “the intended usage in line with the therapeutic regime” [4] in which social support is a prominent component. The basis is a recently finalized RCT in which Web-based support was offered to women with type 1 diabetes mellitus (T1DM) in pregnancy and early motherhood: the MODIAB-Web (MOtherhood and DIABetes) study.

The following research questions have been elaborated upon in this paper:

- What persuasive content and technological elements were used in the design of the Web-based support?
- How was the Web-based support used?
- What was the content of the social peer support and how did the peers support each other?
- What were the main challenges in relation to adherence to the RCT?

## Methods

### Research Design

Using an exploratory case study methodology [6] and the framework for analysis offered through the PSD model [5], we have critically examined the MODIAB-Web study. The analytical focus was on technology and its design, as well as the usage of the different parts of the Web-based support with special focus on the forum for peer support.

### The Motherhood and Diabetes (MODIAB)-Web Study

The MODIAB-Web RCT study was directed at women with T1DM registered at 6 hospital-based antenatal care units in Sweden. Complementary Web-based support was offered to the intervention group, with support starting in early pregnancy and persisting up to 6 months after the child was born [7,8]. The rationale for the study was that T1DM women face particular demands in relation to pregnancy, childbirth, and early motherhood. During pregnancy, they struggle to achieve normal blood glucose levels, which optimize the probability of giving birth to a healthy child [9-11]. In the early months after childbirth, the women have to deal with unstable blood glucose levels, whereas at the same time having to meet all the challenges of early motherhood, and especially those related to breastfeeding [9,12,13]. Their need of Web-based support including social peer support has been identified in earlier studies [14-16].

The Web-based support was developed in line with the ideal of participatory design [17] and includes three parts: (1) information based on scientific evidence, (2) a self-care diary, and (3) a forum for peer support divided into three topics: pregnancy, childbirth, and life as a new mother. In addition, the Web-based support comprised a frequently asked question section where participants could ask questions and receive answers from experts in the field, and a collection of links to other recommended resources. The intention was to offer complementary Web-based support as an add-on to regular pregnancy care and use it especially after childbirth to fill the gap of healthcare support between the different healthcare providers, that is, the maternity care professionals and the diabetes care professionals. If they wished, the women could share their self-care diaries with healthcare professionals during their visits, as the diaries were part of the Web-support structure [7]. The hypothesis was that the Web-based support should strengthen the women’s personal capacity and autonomy, thereby leading to improved self-management of diabetes and overall well-being. Ethical approval was attained from the Ethics Committee of Gothenburg, Sweden (No. 659-09), and the trial was registered at clinicaltrials.gov (ID: NCT015665824). Eligible study participants were successively recruited by an appointed study midwife in early pregnancy. The study was performed over a period of more than 4 years. The first study participant was included on November 22, 2011, and the last participant ended participation on January 25, 2016, that is, 6 months after the last childbirth. Further details about the study design are described elsewhere [7,8].

The findings showed that the Web-based support plus standard care was not superior to standard care alone in terms of general well-being and self-efficacy of diabetes management. Details on this are reported elsewhere [18].

### Analysis

Research question 1, “*what persuasive content and technological elements were used in the design of the Web-based support?,”* has been answered using the PSD model for coding design principles in different categories, each one comprising 7 design principles. The categories are: primary task support, dialogue support, system credibility support, and social support [5]. A deductive analysis consisted of identifying and describing which PSD principles and technological elements were used in the MODIAB-Web intervention. Research question 2, “*how was the Web-based support used?*”, was answered using descriptive statistics and qualitative content analysis [19]. Research question 3, “*the content of the social peer support and how the peers supported each other”*, was answered using descriptive statistics and qualitative content analysis [19]. Initially, the forum posts were read as a whole several times to get an overall sense of the data. In the next step, new readings followed in which the data were organized. This process included open coding, in which notes and headings were written in the margins. The headings were transferred to a separate coding sheet and were grouped into categories. The categories were presented under the preset topics of pregnancy, childbirth, and life as a new mother. The quotations used to illustrate the dialogue in the forum for peer support were professionally translated into English. The analysis was performed by MB in close collaboration with AA. Research question 4, “*what were the main challenges in relation to adherence to the RCT?*”, was answered through a critical analysis based on the results of the first three research questions and the intervention as a whole.

## Results

### Persuasive Content and Technological Elements in the Design of the Motherhood and Diabetes -Web Support

The implemented categories with used design principles [5] in the MODIAB-Web study are presented in Table 1. In the category *Primary Task Support* the focus is on providing technological elements to manage the targeted behavior [5]. The MODIAB-Web study implemented 3 out of 7 design principles: tailoring, reduction, and self-monitoring*,* whereas tunneling, personalization, simulation, and rehearsal were not used. In *Dialogue Support* the focus is on providing various kinds of feedback between the human and the system [5]. In the MODIAB-Web study reminders, liking and social role were implemented, whereas praise, rewards, suggestions, and similarity were not. In *System Credibility Support*, the design principles aim to increase credibility and consequently to be more persuasive [5]. Five design principles were used in the MODIAB-Web study: trustworthiness, expertise, surface credibility, real-world feel, and authority, whereas third-party endorsement and variability were not. The category *Social Support* motivates usage by leveraging social influence [5]. The MODIAB-Web study implemented the principles social learning and social comparison while the principles normative influence, social facilitation, cooperation, competition, and recognition were not used.

### Use of the Motherhood and Diabetes Web-Based Support

**Figure 1:**
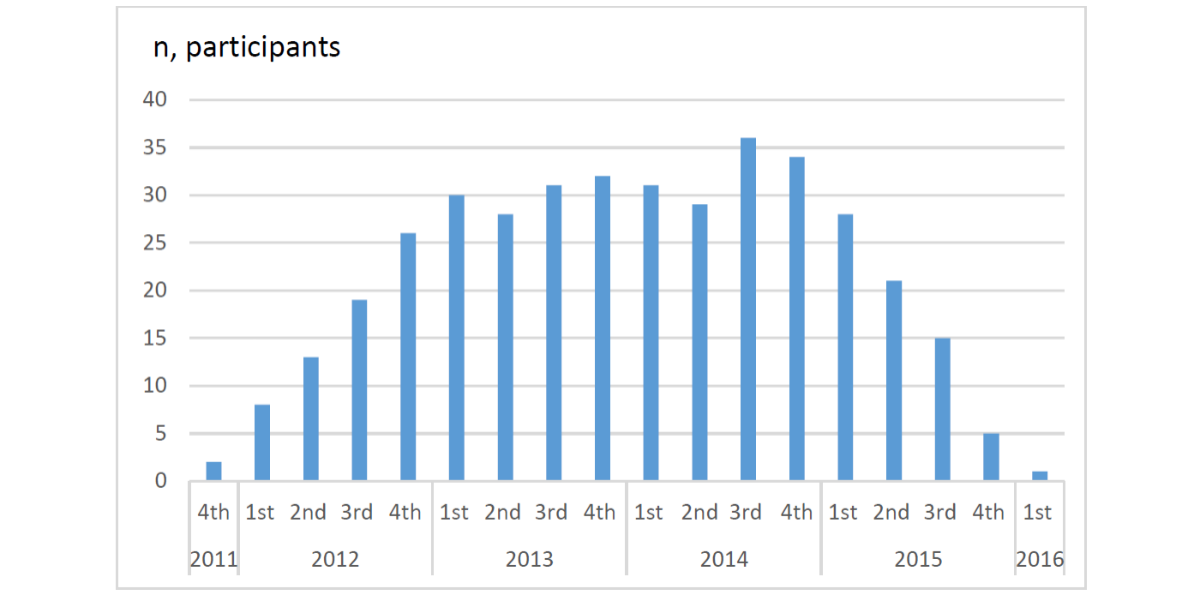
Possible active users during the study period of the MODIAB-Web intervention.

In total, 83 women were randomized to the MODIAB Web-based support, and of these, 81 received a log-in. As the women were successively randomized to either control group or intervention, and as the intervention group could use the Web-based support from randomization to 6 months after birth of the child, a maximum of 36 women could be active simultaneously. This is illustrated in Figure 1.

Of the 81 women with log-in, 69 women were classified as “active users,” that is, they had logged in at least once after the introduction session. How the participants used the Web-based support is presented in Table 2.

The peer support forum was used by 63 women of whom one was a facilitator appointed by the researchers in the first year of the intervention. This facilitator was an experienced mother with T1DM. After that period, it was presumed that the included women could collectively run their activities. The forum was moderated by a member of the research team to have some kind of control over what was discussed and to make sure it did not include advice that was contrary to current scientific evidence. Almost all forum activity occurred in the first 3 years (2011-2014). Of the 63 forum users, 39 participants were readers and 24 were active writers including the study facilitator.

### Forum Content and Type of Peer Support

There were 109 written posts (range: 1-20) divided into 19 threads, of which 84 posts (16 threads) were related to the topic *pregnancy*, 15 posts (1 thread) to *childbirth*, and 10 posts (2 threads) to *life as a new mother*. No inappropriate advice was given during the study period, so the moderator did not have to act. Some women who asked questions in their posts had to wait some time before receiving an answer, while others did not receive an answer at all. This happened after the study facilitator had left the forum.

#### Forum Content

The design of the forum for peer support was intended to help users sort experiences in relation to the different phases of pregnancy, childbirth, and life as a new mother. To some extent, it became apparent that the topics overlapped in each category.

**Table 1:** Categories and design principles in the Motherhood and Diabetes (MODIAB)-Web intervention according to the Persuasive Systems Design (PSD) framework model.

Category and design principle^a^			Implementation in the MODIAB-Web intervention
**Primary task support**			
	**Tailoring**		
		Information provided by the system will be more persuasive if it is tailored to the potential needs, interests, personality, usage context, or other factors relevant to a user group [5].	The evidence-based information was tailored to three themes [20], adapted to reflect the unique aspects that type 1 diabetes adds: being pregnant; labor and childbirth; and life as a new mother
	**Reduction**		
		A system that reduces complex behavior into simple tasks, helps users perform the target behavior, and may increase the benefit/cost ratio of a behavior [5].	The evidence-based information, presented with headlines and clickable subheadings to scan for quick access, contained simple task helps for the participants such as what a healthy breakfast might consist of and how to adjust insulin doses in the first days after childbirth [20].
	**Self-monitoring**		
		A system that keeps track of one’s own performance or status and supports the user in achieving goals [5].	In the self-care diary, the women could use either a smartphone or computer to register daily life information, such as blood glucose levels, insulin and food intake, and overall well-being status. The registered data were presented in tables and diagrams, with the intention of supporting the woman in analyzing and managing her daily life to accomplish optimal blood glucose levels. This information could also be presented to health care professionals if the women consented.
**Dialogue support**			
	**Reminders**		
		If a system reminds users of their target behavior, the users are more likely to achieve their goals [5].	Text messages to inactive users were sent to the participants as reminders every 2 weeks consisting of a greeting and contact information in case of technical difficulties. There was no flagging of new posts in the forum for peer support.
	**Liking**		
		A system that is visually attractive to its users is likely to be more persuasive [5].	The Web-based support was developed in collaboration between the research group and Web designers and evaluated by a group of mothers with type 1 diabetes [21]. Pictures of pregnant women and babies were used to illustrate the content.
	**Social role**		
		If a system adopts a social role, users are more likely to use it for persuasive purposes [5].	The system was designed to enable the women to coach each other. During the first year of the intervention when there were very few participants, a “coach,” a woman with type 1 diabetes who had given birth to a child, was used as a social facilitator in the forum. She initiated contact and replied to new users’ posts in the forum.
**System credibility support**			
	**Expertise**		
		A system that is viewed as incorporating expertise will have increased powers of persuasion [5].	All parts of the Web-based support were developed in a collaborative developmental process by researchers and experts; including experienced mothers with diabetes and Web-designers. The professional experts were: nurse-midwives specializing in diabetes care and human lactation; gynecologist/obstetricians; diabetologists; neonatal nurse; dietician [7,20]. The support’s design was reviewed by experienced mothers with type 1 diabetes [21].
	**Trustworthiness**		
		A system that is viewed as trustworthy will have increased powers of persuasion [5].	The names and titles of the healthcare professionals who had contributed to the design were clearly stated in the information section of the Web-based support.
	**Surface credibility**		
		People make initial assessments of the system credibility based on a firsthand inspection [5].	The Web-based support was test-piloted by a focus group consisting of women with diabetes with experience of pregnancy and childbirth. The Web-based support was adapted in line with their comments [21].
	**Real-world feel**		
		A system that highlights people or organization behind its content or services will have more credibility [5].	The frequently asked questions section contained the participants’ questions and the anonymized expert answers, which were published for all to read. There was a delay of up to 2 weeks before answers were received and later published.
	**Authority**		
		A system that leverages roles of authority will have enhanced powers of persuasion [5].	The physician-in-chief of the respective antenatal care units supported the intervention. This was clearly stated in the Web-based support. The system did not include health care professionals as users.
**Social support**			
	**Social learning**		
		A person will be more motivated to perform a target behavior if he can use a system to observe others performing the behavior [5].	In the forum for peer support, the users could start up their own threads as an opportunity for learning from each other’s experiences under the three defined themes: pregnancy, childbirth, and life as a new mother.
	**Social comparison**		
		System users will have a greater motivation to perform the target behavior if they can compare their performance with the performance of others [5].	The system partly supported social comparison between participants, specifically in the forum for peer support in which experiences were shared.

^a^Cited from the description of the PSD model by Oinas-Kukkonen and Harjumaa [5].

**Table 2:** Web-based support usage.

Activities	N	Median	Minimum	Maximum
Total log-ins by active users	69	91	2	6413
Evidence-based information	61	10	1	508
Forum for peer support	62^a^	60	1	703
Self-care diary	37	21	1	5850
Frequently asked questions	53	2	1	17

^a^A study facilitator participated in the first year, giving 63 active users.

##### Pregnancy

This topic had the largest number of posts. Its content related to overly focusing on diabetes as a disease; the first ultrasound; how to handle glycemia and specifically hypoglycemic episodes; insulin dosages, and the estimated weight of the expectant child. Minor discussions concerned managing attendance at the many checkups combined with working and how to plan for parenthood after the birth of the child, including how to get in contact with the Swedish social insurance agency for the parental benefit.

##### Childbirth

The posts related to inducing labor and labor routines, including how the insulin was administered and if an insulin pump was permitted during labor. Other posts discussed wounds and wound healing. One woman who had given birth 3 times shared her story of all these childbirths, one having been vaginal, one vaginal vacuum extraction, and one elective cesarean section.

##### Life as a New Mother

Participants shared posts on this topic for a period of only 6 months (December 2011 to July 2012). Content mostly concerned postpartum care at the hospital—that there was insufficient professional support or insufficient knowledge about diabetes. Another main concern was breastfeeding-related issues. One woman expressed her disappointment that the ward ignored her wish to avoid giving her baby cows’ milk-based nutrition supplements. Another woman concluded that in her situation with diabetes, it was best to combine breastfeeding and a milk supplement. One woman asked for experiences of breastfeeding and how to integrate it in daily life as a mother with diabetes. Unfortunately, no one responded. In addition, there were many diverse posts, for example, about a baby who had got an infection, and another baby who had a clavicle fracture. Several questions were asked, for example, did you get preeclampsia? What type of pain relief did you choose? One woman, who was active at the beginning of the intervention period (December 2012), advertised her own blog and its weblink.

#### Type of Support

The analysis of the peer support in the forum shows that, although there were few participants there was a rich exchange between them. The dialogues comprised sharing of personal experiences, giving concrete advice, affirmations, and words of reassurance. Examples of two dialogues between the participating women are given in Textbox 1.

**Examples of dialogues in the peer forum between participating women.**

**Dialogue—Example 1**

Woman 1: “As a diabetic, having kids isn’t a walk in the park but I’ve managed twice before with completely perfect kids…this pregnancy has been really tough – with high blood pressure and low sugar but after a lot of messing about, it’s sorted itself out now.”

Woman 2: “I’m so inspired and motivated when I hear people like you – congratulations on pregnancy number three! I’m in my first but we’re almost the same age and I’ve been a diabetic for 22 years. My biggest worry is the hypos. I hate them and I worry the baby can feel them. Once all its organs are ʻI’m s’ and I know it can produce insulin and has a liver with a glycogen store, I’ll be calmer. My levels aren’t low all the time – my HBA1C is 7.9 so it has to be lowered. One side effect of my managing to lower it is that I’m more insulin sensitive. But I know that changes in different stages of pregnancy. Was it obvious in your other pregnancies – in which case, when might you have become more insulin resistant?

I’m noticing I’m actually pregnant now, week 10+4, and I’ve done the CUB (Combined Ultrasound and Biochemistry screening) and one ultrasound. So it’s weird and wonderful to see that a fetus is living inside me. Of course, it’s early yet but it feels real now. I won’t tell them at work until after week 12. How do you feel about telling people, when did you do that?

Woman 1: “Yes the hypos suck, I have to say but don’t worry during the first weeks, during this pregnancy I had such low levels an ambulance had to come lots of times but those little ones are tough – nothing happened to him. Now I’m in week 20+. What happens when you have high levels is that the baby has to work harder to produce more insulin but at low levels, not much happens, as long as you don’t go into a coma, that is – don’t push yourself or you’ll go mad. I told work about my first pregnancy straight away but that was because I had heavy lifting and stuff to do. I’ve always felt you should say when you’re ready to say you’re going to become a mum. And it takes time, I promise you – I’ve been a mum for almost eight years now and I still can’t see myself as one. Everything will be alright, you’ll see. Everything’s tough at the start but with time it gets better and calmer.”

Woman 2: “Thanks for such a good answer – it’s calming what you write about having hypos.”

**Dialogue—Example 2**

Woman 3: “I wonder if there are others like me who think it feels like all the focus is on the disease/s and that you never talk about the pregnancy. I’m in week 29 and had diabetes for 22 years and I also have trouble with my thyroid. My blood sugar and thyroid tests all have great results but still the midwife just keeps talking about the diseases. Have you experienced that? Do you get enough support around pregnancy issues or is the focus only on the diabetes?”

Woman 4: “I think that’s varied a bit with each of my different pregnancies. It’s had a lot to do with the midwife I had. Sure, there’s been a lot of focus on diabetes and blood sugar levels but the midwife I have now makes a point of also talking about the baby and pregnancy-related things. But my last one wasn’t like that…I think you should raise it with your midwife again – say you need her help with some pregnancy issues. After all, she’s there for you and your baby! Good luck!”

Woman 3: “Thanks! I’ll try again tomorrow!!”

Woman 5: “I agree the focus is mostly on the diabetes when you visit the midwife – you almost forget what you’ve been wondering about and want to discuss with the midwife but the only thing they want to talk about is test results. Of course, it’s good to know they’re really on the ball with the diabetes during the pregnancy but you’ve never felt this ill as a diabetic after living with it for so many years.”

### What Were the Main Challenges in Relation to Adherence to the Web-Based Randomized Controlled Trial?

This section comprises a critical analysis of both significant and minor issues in relation to adherence (defined as “the intended usage in line with the therapeutic regime” [4]) to the Web-based intervention. It is also based on the fact that the presumed effect of the RCT, in terms of increased well-being and diabetes management, was not achieved [18]. The MODIAB-Web study was typically designed according to the gold standard of an RCT [22]. How did this come about and why?

#### Adherence in Relation to Study Design

There were few women available to include in this study. In Sweden, around 0.5% of all pregnant women have T1DM, corresponding to 500 per year. At the beginning, two study centers were included based on an estimate of few eligible women. It became apparent that the recruitment rate of study participants was slower than expected. In addition, four study centers were included to avoid a more prolonged data collection period. Furthermore, the contact frequency between the researchers and the healthcare professionals at the study centers was increased. Prolonging clinical trials because of slow recruitment pace and retention of study participants is a common issue. One reason for this might be poor engagement from the healthcare professionals who are inviting the patients to take part in the study [23].

The slow inclusion pace prolonged the study duration, which in turn meant only a few women (about 30) could be active at the same time (Figure 1). Thus, the critical mass of simultaneous active users was not sufficient and undermined the extent of interactions in the Forum. It has been concluded that many of the participants in a Web-based study may be lost to follow-up or end up not adhering to the intervention [2]. This was not taken into account when calculating the sample size. One way to get around this could have been to have started the study at more study centers from the beginning to gain a greater critical mass faster.

The use of a facilitator who was active in the peer support forum the first year was fruitful, as it increased the dialogues in the peer support forum. A shortfall in the study design was that the facilitator was not engaged during the whole study period, which led to the activity level in the forum dropping.

The quality of the support shared between the participants in the MODIAB-Web study was reasonably good, as exemplified in the dialogues (Textbox 1). The active writers in the forum for peer support coached each other and shared their experiences in a generous way. The majority of participants were “readers”; they did not actively participate in the dialogues. However, in line with recent research, these are probably also “passive actors”—meaning only readers can receive social support related to their specific needs [24].

The forum user who advertised the weblink to her blog in the MODIAB-Web study at least potentially created an alternative Web-based support. This makes it hard to truly evaluate the usefulness of the intervention, as meaningful interaction might happen outside the study platform. It also proposes a threat to adherence to the intervention, if participants choose to communicate in alternative ways, such as through social media. It is almost impossible to control for this, and it is a methodological weakness in studies evaluating Web-based social support.

A main problem arising due to the prolonged duration of the MODIAB-Web RCT study was the simultaneous explosion of more advanced general mobile phone technology [24] as well as similar technology in diabetes care contexts. The developed Web-support serving as a platform for self-management consequently became outdated with time as few available alternatives grew.

#### Adherence in Relation to Technology

With regard to the PSD model [5], four categories were identified in the MODIAB-Web intervention: *Primary Task Support*, *Dialogue Support*, *System Credibility Support,* and *Social Support* (see Table 1)*.* This shows that several but not all of the design principles were implemented*.*

To accomplish adherence, Kelder et al argue that it is essential to actually plan for adherence when designing Web-based interventions [4]. The MODIAB-Web intervention was developed using participatory design [17] and grounded on previous empirical and theoretical investigations of the needs of the user groups, as well as ongoing user participation of lay and professional experts [7]. However, there was no theoretical basis for the technological design or the use of design principles of a more general kind, such as the PSD model. A theoretical grounding would have generated several advantages. One prominent advantage is insights into a greater repertoire of design elements than those available through participating lay and professional experts. In this manner, design is based on practical and theoretical knowledge outside of the local project.

Moreover, a theoretical grounding such as the PSD model was launched in 2009, meaning that it existed well before the investigations of general user needs [14] and the design of the MODIAB-Web intervention in particular [7]. However, it must be recognized that the recent increasing importance and subtleties of research processes focusing on design to accomplish behavior change are still treated in specific research fields such as design studies [25]. A theoretical grounding when it comes to behavior change itself would enable a repertoire of more passive and active strategies for influencing people that, in turn, should be brought into the technology design part of the intervention. Such strategies might involve using models of how to accomplish a feeling of social connectedness [26], which is relevant in interventions aiming to increase the social support between peers. A theoretically more conscious design might be an alternative to the recent suggestion of generating predictive models of potential dropouts in online health communities by Big Data analysis to inform design [27].

The use of design theory in the analysis of the MODIAB-Web intervention enabled a discussion of the rationale behind its emphasis on *System Credibility Support* and its significantly less emphasis on *Dialogue Support* and *Social Support*. Quite likely, potential was lost in unutilized design principles. For example, in the *Dialogue Support* category, the design principle “Rewards” that aims to give credit for performing the target behavior [5], that is, using the Web-support, could have been implemented to increase adherence. For instance, this could entail rewarding participants once they had used the support 5 times, or when at least 5 other participants had read a person’s posts in the forum.

A further technical element related to *Social Support*, not used in the MODIAB-Web forum for peer support, was “flagging” of new posts. Such flagging had probably increased the participants’ “adherence,” in terms of active communication. Another issue that probably reduced adherence was that the forum was divided into 3 parts. This division meant, there was a risk, that new posts were not seen by the other participants. Another shortfall was that the participants were not obliged to write a post to introduce themselves upon first entering the peer support forum. Admittedly, certain PSD design principles related to *Social Support* would have been positive in relation to adherence, for example, “Recognition,” which would include success stories. Other design principles, such as “Normative influence,” might be less relevant in this context due to the extreme demand for optimal diabetes control for childbearing women.

## Discussion

In this paper, we used case study methodology to enable a critical discussion of RCTs involving Web-based support and adherence in general, and social support in particular. In other words, this methodology has been used as a basis for our critical endeavor in line with Baxter and Jack, who state: “it enables the researcher to answer ‘how’ and ‘why’ type questions, while taking into consideration how a phenomenon is influenced by the context within which it is situated” [6].

In this paper adherence is, in line with Kelders et al [4], defined as “use in accordance with identified intentions,” and in which the need for relevant information about the specific condition as well as social support from peers is emphasized. Our analysis supports the conclusion that PSD does matter in general, by positively influencing adherence [4]. We argue that there is a particular value in basing technology design on theoretical design principles, as it increases the repertoire of options, thus enabling adaption to suit the target population for the intervention and creating opportunities to utilize the full design potential of social support. The value of applying theory ties in with recent acknowledgment of theoretical consciousness regarding adaptation and implementation of health information technology innovations [28]. When it comes to health information technology such as Web-based support, we argue that theories related to technology use and design, such as the ones discussed above, are equally important. This notwithstanding, the essential rationale of Web-based health interventions remains the same but becomes significantly better theoretically informed. A systematic review published post completion of our analysis showed that justifications for intended use, and adherence to eHealth technology is often underdeveloped and improperly used. The authors believe that adherence can be standardized, and this will improve comparison of adherence rates to different technologies with the same goals [29]. Our critical analysis thus contributes to ongoing discussion about adherence.

Moreover, we conclude that study design matters and that technology and study design might mutually influence each other. With a limited target group, the comparative element that constitutes an RCT design becomes a barrier to achieving a critical mass of user interactions. Extended project periods mean that the used technology must either be changed or tailored according to new technical options that become available during the process, which in turn might jeopardize study validity. Not doing so implies a risk that the technology will become somewhat outdated in the world of the users. In relation to providing social support, a further conclusion is that using theory enables a multifaceted repertoire in technology design. However, we also note that apparently low user activity might still provide social support for the individual, irrespective of whether they are an active or passive user.

On the basis of critical analysis of how study design and technology interacts, we recommend fellow researchers to consider the following aspects when designing and evaluating Web-based health interventions:

- When designing a Web-based intervention, use existing design theories to utilize the full potential of the technology and increase adherence, especially with regard to social support.
- Be realistic when calculating your sample size. Take the risk of losing participants and poor adherence to the intervention into account.
- Be aware of limited target populations. If the number of possible participants is low, consider starting the intervention in multiple study centers simultaneously to avoid prolonged study periods and thereby outdated technology.
- Bear in mind that the majority of participants will not actively engage in discussions in peer support forums. Therefore, a larger critical mass of participants is needed in interventions based on effects of social support.
- Keep the design of the forum for peer support simple and avoid divisions. Web-design is often costly if the researchers do not possess the skills themselves or team up with researchers from other fields. Consider integrating your intervention into existing social media (but be aware of ethical pitfalls if you cannot guarantee the security and confidentiality of data).
- If your intervention consists of a forum for peer support, consider engaging a study facilitator from the target group for the entire study period to boost activity.
- Health interventions are often complex in their nature. RCT design is one way of evaluating the effects of an intervention but it is in itself inadequate for truly capturing complex interactions. Design your study with multiple methods of analysis and consider the contamination of the control group as technology evolves during the study period.
- Adjust per-protocol criteria after intended usage or conduct a dose-response analysis to properly evaluate the effect of the intervention within the RCT design.

Finally, we want to return to the initial critical questioning of whether the RCT design really is appropriate as the gold standard for Web-based interventions [2]. Our analysis does not fully answer this question, nor does it reveal what could be a better research design, but we would like to encourage further constructive discussion on these issues within the scientific community.

## Abbreviations

MODIAB-Web: Motherhood and Diabetes Web
PSD: persuasive systems design
RCT: randomized controlled trial
T1DM: type 1 diabetes mellitus
